# Exploring protein *N*-glycosylation in ammonia-oxidizing *Nitrososphaerota* archaea through glycoproteomic analysis

**DOI:** 10.1128/mbio.03859-24

**Published:** 2025-05-19

**Authors:** Satoshi Nakagawa, Hirokazu Yagi, Tomoki Suyama, Shigeru Shimamura, Saeko Yanaka, Maho Yagi-Utsumi, Shingo Kato, Moriya Ohkuma, Koichi Kato, Ken Takai

**Affiliations:** 1 Laboratory of Marine Environmental Microbiology, Division of Applied Biosciences, Graduate School of Agriculture, Kyoto University 220883 , Kyoto, Kyoto Prefecture, Japan; 2 Super-cutting-edge Grand and Advanced Research (SUGAR) Program, Institute for Extra-cutting-edge Science and Technology Avant-garde Research (X-star), Japan Agency for Marine-Earth Science and Technology (JAMSTEC) 13570, Yokosuka, Kanagawa Prefecture, Japan; 3 Exploratory Research Center on Life and Living Systems (ExCELLS), National Institute of Natural Sciences, Okazaki, Aichi Prefecture, Japan; 4 Graduate School of Pharmaceutical Sciences, Nagoya City University 12963, Nagoya, Aichi Prefecture, Japan; 5 Institute for Molecular Science (IMS), National Institutes of Natural Sciences 13592, Okazaki, Aichi Prefecture, Japan; 6 Japan Collection of Microorganisms (JCM), RIKEN Bioresource Center 375695 , Tsukuba, Ibaraki Prefecture, Japan; University of California Irvine, Irvine, California, USA

**Keywords:** glycan, *Nitrososphaerota*, ammonia-oxidizing archaea, *Thaumarchaeota*, S-layer

## Abstract

Ammonia-oxidizing archaea of the phylum *Nitrososphaerota*, formerly known as *Thaumarchaeota*, are globally distributed and play critical roles in the nitrogen and carbon cycles, particularly in environments with low ammonia concentrations. Like most archaea, *Nitrososphaerota* cells are enveloped by S-layer proteins, implicated in concentrating ammonium ions. These proteins are typically modified post-translationally by *N*-glycans, which often play significant roles in various biological processes, including protein function regulation, protection from phages, and environmental adaptation. Nevertheless, the glycobiological characteristics of *Nitrososphaerota* remain largely unexplored. Here, we investigated the glycoproteome of ammonia-oxidizing *Nitrososphaerota*, specifically focusing on the terrestrial *Nitrososphaera viennensis* and the marine *Nitrosopumilus piranensis*. Both species exhibited similar protein arrays throughout their growth phases, including those associated with *N*-glycosylation. *Ns. viennensis* consistently exhibited *N*-glycosylation predominantly on an S-layer protein and multicopper oxidase domain-containing proteins throughout all growth phases, with a marked increase during and after the late exponential phase. The glycan, characterized as a novel hexasaccharide with a chitobiose core, is hypothesized to play a role in nitrogen storage due to its probable nitrogen-rich composition, modifying asparagine residues within the conserved triplet sequence (Asn-X-Ser or -Thr). In contrast, *Np. piranensis* also showed a high abundance of S-layer protein but displayed no apparent *N*-glycosylation on any protein, suggesting variability in cell surface physical properties between these archaea. Despite similarities in their proteomes and energy metabolism, these two archaea exhibited significant differences in post-translational modification of proteins, revealing previously unrecognized diversity that may have implications for understanding their adaptive transitions to diverse environments.

**IMPORTANCE:**

Autotrophic ammonia-oxidizing archaea of the phylum *Nitrososphaerota*, formerly known as *Thaumarchaeota*, are notoriously difficult to culture yet play important roles in the global nitrogen and carbon cycles. Inhabiting environments with extremely low ammonia concentrations, these archaea are expected to conserve ammonia strictly for energy production. However, using advanced liquid chromatography-tandem mass spectrometry and nuclear magnetic resonance techniques, we discovered that one of these archaea decorates its cell surface proteins with the most nitrogen-rich glycan identified to date, suggesting a previously unrecognized function of protein glycosylation in nitrogen storage. This newly identified *N*-glycan, with a chitobiose core similar to those in *Thermoproteota* and eukaryotes, not only deepens our understanding of archaeal evolution but also underscores the molecular adaptations enabling these archaea to thrive in diverse environments.

## INTRODUCTION

Members of the phylum *Nitrososphaerota*, formerly known as *Thaumarchaeota*, are predominantly recognized as ammonia-oxidizing archaea (AOAs) (1). Initially identified through culture-independent analysis in coastal marine environments, these archaea were designated as Marine Group I (2, 3). They have been detected throughout oxygenated marine environments and even in symbiotic relationships within a marine sponge (4). Additional detections have been reported from terrestrial habitats including soils (5, 6), lakes (7), and hot springs (8). The first pure culture, *Nitrosopumilus maritimus*, was isolated from a seawater aquarium (9). Following this isolation, various strains and enrichments from both terrestrial and marine environments have been reported, all capable of autotrophic growth via ammonia oxidation under aerobic conditions (10–22). Considering their ubiquity, metabolic activity, and high affinity to ammonia at low concentrations (23, 24), these archaea play important roles in the global nitrogen and carbon cycles and in greenhouse gas production (1, 25–31). Some deeply branched members of *Nitrososphaerota*, such as *Conexivisphaera calidus*, are heterotrophic rather than AOA (32–34), indicating metabolic variability within this group of archaea. Terrestrial *Nitrososphaerota* generally possess larger genomes compared to their marine counterparts (35, 36). Genome expansion within the terrestrial *Nitrososphaerota* was probably driven by lateral gene transfer and extensive gene duplication (36). Additionally, the habitat expansion of *Nitrososphaerota* is probably influenced by lateral gene transfer, particularly involving the ATPase gene (37). Significant geological events, such as glaciation and oxygenation, may have stimulated the evolutionary transition of these microorganisms from terrestrial to oceanic environments (38, 39). In addition, differences in response to copper limitation were observed between terrestrial and marine strains (40). However, further exploration of these differences is needed to understand their adaptive transitions to diverse environments.

Previous proteome analysis of *Nitrososphaera viennensis* revealed an abundant occurrence of S-layer proteins (35), consistent with earlier findings that S-layer proteins comprise 10%–30% of the total cellular protein content in archaea (41). Electron microscopy has confirmed the presence of the S-layer; however, the protein symmetry varied among *Nitrososphaerota*. Specifically, the terrestrial *Ns. viennensis* exhibited a p3 symmetry in its S-layer protein array (42), similar to those observed in *Sulfolobales* spp. (43). In contrast, the marine *N. maritimus* displays the typical p6 symmetry observed in diverse archaeal species (21). Although the significance of these crystallographic differences is not yet fully understood, they imply variations in cell surface properties among AOA. These variations may reflect their adaptations to different environments (44), despite their shared capability to oxidize ammonia. In *N. maritimus*, it was suggested that S-layer proteins with cation-binding properties play a role in nutrient capture (45).

Archaeal S-layer proteins are typically modified by *N*-glycans, which serve a variety of biological functions (46, 47). Although previous genomic analyses have identified some genes associated with protein *N*-glycosylation in the genomes of *Nitrososphaerota* (35, 48, 49), specific glycobiological characteristics such as the diversity of glycosylated proteins, glycan structures, and glycosylation sites remain unknown. Archaeal S-layer glycosylation often plays an adaptive role by altering cell surface properties to provide protection, enhance survival under changing environmental conditions, and facilitate cell-cell interactions (47, 50), and thus may contribute to the habitat expansion of *Nitrososphaerota*. Additionally, the structure of archaeal *N-*glycans, including the composition and sequence of sugars, varies significantly across the phylogenetic tree, with each archaeal species producing its own unique *N*-glycan (46). For instance, the structure of *N*-glycans in members of *Thermoproteota*, such as *Sulfolobus* and *Pyrobaculum*, which feature a chitobiose core at the reducing end similar to that of eukaryotes, provides insights into archaeal contributions to eukaryogenesis (51–54). Currently, no glycan structures have been characterized for *Nitrososphaerota*. The poor growth of *Nitrososphaerota* has restricted the use of conventional glycobiological methods. However, recent advancements in mass spectrometry technology, combined with open modification search methods, have facilitated glycoproteomic analyses using small amounts of biomass from fastidious prokaryotes (55), as evidenced in our previous studies (54, 56). While mass-based analysis has its limitations, such as the inability to distinguish stereo-isomers, these technological advances have expanded our understanding and would direct our research toward specific aspects or species that warrant further detailed investigation.

In this study, we performed a glycoproteomic analysis on two species of *Nitrososphaerota*, *Ns. viennensis* (11, 42) and *Np. piranensis* (20). We discovered significant differences in their glycobiological profiles. This study provides new insights into the ecophysiological diversity within this important group of archaea, enhancing our understanding of their adaptive mechanisms and evolutionary processes.

## MATERIALS AND METHODS

### Strains and cultivation

*Ns. viennensis* strain JCM19564 (=EN76^T^) and *Np. piranensis* strain JCM32271 (=D3 C^T^) were cultivated without agitation in 3,000 mL flasks containing 1,500 mL of medium, following the guidelines of the Japan Collection of Microorganisms. Cell growth was assessed through direct cell counts after staining with 4′6-diaminido-2-phenylindole under a fluorescent microscope (57). Nitrite concentration in the cultures was measured using a digital PACKTEST (dpm2-no2; Kyoritsurikagaku, Tokyo). Cells were harvested by centrifugation from 450 mL cultures at the exponential, late exponential, and stationary phases for further analysis.

### Protein extraction and trypsin digestion

Proteins were extracted using a method described previously (58). Briefly, cells were resuspended in 100 mM triethylammonium bicarbonate at pH 8.6, containing 2 mM phenylmethylsulfonyl fluoride, and disrupted by sonication at 2°C. A total of 20 µg of protein pellet was dissolved in MPEX PTS reagent (GL Science, Tokyo, Japan), then reduced with dithiothreitol, and alkylated with iodoacetamide. The samples were digested with trypsin (Thermo Fisher Scientific, Waltham, MA) and lysyl endopeptidase (Fujifilm Wako, Osaka, Japan) (56). Detergents were removed using a spin column (Thermo Fisher Scientific), after which the peptide samples were subjected to liquid chromatography-tandem mass spectrometry (LC-MS/MS) analysis.

### LC-MS/MS analysis

Peptide samples were separated using an Ultimate 3000 RSLCnano system (Thermo Fisher Scientific), as previously described (59). The samples were introduced onto the trap column using a sample buffer (0.1% trifluoroacetic acid, 2% acetonitrile) and subsequently eluted into an Orbitrap Fusion Tribrid Mass Spectrometer (Thermo Fisher Scientific) at a flow rate of 500 nL/min (buffer A, 0.1% formic acid) through the analytical column. The analytical runs lasted 230 min, with the buffer composition initially transitioning from 5% to 45% vuffer B (100% acetonitrile) over 225 min, and then from 45% to 95% vuffer B in the final 5 min.

The mass spectrometer was operated in positive ion mode as previously described (54, 56). MS spectra were collected in the Orbitrap mass analyzer (*m*/*z* range: 350–1,800, resolution: 120,000 full width at half maximum with a maximum injection time of 50 ms, automatic gain control [AGC] 5 × 10^4^) using EASY-IC for internal mass calibration. Orbitrap tandem mass spectrometry (MS/MS) higher-energy collision dissociation (HCD) scans of precursors involved parameters such as NCE 28%; maximum injection time set to auto; AGC 5 × 10^4^ at a resolution of 50,000; and an isolation window of *m*/*z* 2. To minimize repeated analysis of identical components, a dynamic exclusion period of 60 s was implemented. Specific oxonium ions, including HexNAc at *m*/*z* 204.087 and its fragment at *m*/*z* 138.0545, triggered two additional scans for potential glycopeptides: an Orbitrap electron-transfer/high-energy collision dissociation (EThcD) scan (NCE 25%; maximum injection time of 86 ms; AGC 5 × 10^4^ with a resolution of 50,000; isolation window of *m*/*z* 3) and a stepped collision energy HCD scan (NCE at 10%, 25%, and 40%; maximum injection time of 86 ms; AGC 5 × 10^4^ at a resolution of 50,000; and isolation window of *m*/*z* 2).

### Data analysis

The LC-MS/MS raw files were analyzed using Byonic software v.3.11.3 (Protein Metrics Inc., Cupertino, CA), a robust tool for identifying peptides and their modifications, as previously described (54, 56). The software facilitates comprehensive protein identification by matching mass spectra against a database, with advanced capabilities for detecting post-translational modifications (60). Genome sequences of *Np. piranensis* and *Ns. viennensis* were sourced from accession numbers NZ_CP010868 and NZ_CP007536.1, respectively. Cleavage specificity was configured as semi-specific N-ragged, allowing up to three missed cleavage events. Carbamidomethyl was set as a fixed modification for cysteine, while methionine oxidation and acetylation of protein N-terminal amino groups were included as variable modifications. Maximum mass precursor tolerance was set to 10 ppm, with mass tolerances of 10 ppm for HCD fragments and 20 ppm for EThcD fragments. For *N*-glycosylation open searches, the wildcard parameter was enabled, allowing a delta *m*/*z* range of 300–2,000 on asparagine residues. The open search capability of Byonic allows for the comprehensive analysis of proteomic profiles and glycan modifications, particularly useful for samples containing unconventional or unknown glycans. This method involves applying a wider tolerance for precursor mass, enabling the identification of modified peptides by assessing the discrepancies between their observed and theoretical masses (60). In focused searches, all parameters remained consistent except for the disabling of wildcard searching, incorporating specific glycoforms identified from open searches as variable modifications. To distinguish bona fide glycan modifications from false positives, criteria were established: (i) the presence of potential sugar oxonium ions in MS/MS spectra, (ii) conformity with the conserved amino acid sequence, and (iii) the occurrence of modifications in peptides generated by proper enzymatic cleavage (54, 56). Protein identifications were filtered with |log prob| of ≥2. Peptide identifications required a Byonic score of ≥200, |log prob| of ≥2, and a two-dimensional false discovery rate of ≤0.01. Relative protein abundances were determined using the normalized spectral abundance factor (NSAF) method, which is a widely accepted method that normalizes spectral counts to protein length, allowing for comparisons across different proteins in a sample (61). Subcellular localization predictions were performed using pSORTb v.3.0 (62). Additionally, *N*-glycosylation sites were predicted using GlycoPP v.1.0 (63).

### NMR analysis

*Ns. viennensis* was cultured in a medium containing 0.2 mM ^13^C-HCO_3_^−^ and 1.8 mM ^12^C-HCO_3_^−^. Glycans were purified according to the method described previously (64) and dissolved in ^2^H_2_O. Two-dimensional total correlation spectroscopy, correlation spectroscopy, nuclear Overhauser effect spectroscopy, ^1^H-^13^C heteronuclear single-quantum coherence (HSQC), and ^1^H-^13^C heteronuclear multiple bond correlation experiments were performed at 298 K using a Bruker AVANCE NEO-800 spectrometer equipped with a cryogenic probe. All NMR data were processed using TopSpin (Bruker, Billerica, MA).

## RESULTS AND DISCUSSION

### *N*-Glycosylation genes

Glycosylation involves various enzymes such as glycosyltransferases and the oligosaccharyltransferase, along with enzymes responsible for biosynthesis and modification of the glycan component sugars (46). To explore the potential for *N*-glycosylation in *Ns. viennensis* and *Np. piranensis*, the gene repertoire associated with this protein modification was examined in both species. Each species possesses a single copy of the oligosaccharyl transferase gene (*aglB*), which catalyzes the last step of glycan transfer to specific asparagine residues and contains the WWDYG motif (49) (Table 1). A BLASTp search, using protein sequences encoded by the *agl* genes of *Haloferax volcanii* and *Methanococcus maripaludis* as queries (46), identified homologous genes dispersed throughout their genomes (Table 1), a pattern typically observed in *Thermoproteota* archaea, with the exception of *Metallosphaera* spp. (49). Although the presence of these genes indicated a potential for *N*-glycosylation in both species, the specific glycoforms or glycosylation sites cannot be determined solely from the genomic data.

**TABLE 1:** Proteins potentially associated with protein glycosylation in *Ns. viennensis* and *Np. piranensis*

Description (query)	Accession no.	agl	*Nitrososphaera viennensis*				*Nitrosopumilus piranensis*			
			Locus tag	NSAF			Locus tag	NSAF		
				96 h	120 h	280 h		120 h	192 h	264 h
Putative acetyltransferase (*Methanococcus maripaludis* S2)	CAF29906.1	17	NVIE_RS03710	0.037	0.043	0.027	NPIRD3C_RS03125	0.026	0.016	0.045
DegT/DnrJ/EryC1/StrS aminotransferase (*Methanococcus maripaludis* S2)	CAF29907.1	18	NVIE_RS11420	0.050	0.052	0.062	NPIRD3C_RS01005	0.014	0.041	0.019
Putative oxidoreductase (*Methanococcus maripaludis* S2)	CAF29908.1	19	NVIE_RS03720 NVIE_RS04905	0.023 ND	0.023 ND	0.033 ND	NPIRD3C_RS03135	0.013	0.025	0.031
UDP-N-acetylglucosamine 2-epimerase (*Methanococcus maripaludis* S2)	CAF29913.1	21	NVIE_RS04355	0.005	0.011	0.004	NPIRD3C_RS00485	0.013	0.004	0.007
Oligosaccharyl transferase, STT3 subunit (*Methanococcus maripaludis* S2)	CAF30980.1	B	NVIE_RS12080	0.044	0.031	0.034	NPIRD3C_RS00730	0.004	0.002	0.007
UTP-glucose-1-phosphate uridylyltransferase AglF (*Haloferax volcanii* DS2)	ADE04323.1	F	NVIE_RS02395 NVIE_RS03290	0.036 0.031	0.044 0.028	0.034 0.033	NPIRD3C_RS08310 NPIRD3C_RS06570	0.035 0.042	0.044 0.041	0.041 0.049
Putative glycosyl transferase, family 4: aldehyde dehydrogenase (*Methanococcus maripaludis* S2)	CAF30979.1	H	NVIE_RS06465	0.003	0.007	0.005	NPIRD3C_RS03115	ND^*a*^	ND	ND
UDP-glucose 4-epimerase related (*Methanococcus maripaludis* S2)	CAF30646.1	W	NVIE_RS10420	0.005	ND	ND	NPIRD3C_RS00115	0.014	0.004	0.008
Similar to bacterial imidazoleglycerol-phosphate synthase, amidotransferase subunit (*Methanococcus maripaludis* S2)	CAF30638.1	Y	NVIE_RS11275	0.030	0.051	0.022	NPIRD3C_RS00435	0.035	0.026	0.048

^*a*^ “ND” indicates not detected.

### Proteomic characteristics

Although proteomic characterization of *Nitrososphaerota* was previously conducted on late exponential phase cells of *Ns. viennensis* (35), early stationary phase cells of “*Candidatus* Nitrosopelagicus brevis” (65), and exponential phase cells of *N. maritimus* (66)*,* this study provides a time-course profile of protein expression. For both strains, cells were harvested at exponential, late exponential, and stationary growth phases for analysis (Fig. S1). In *Ns. viennensis*, our glycoproteomic analysis using an *N*-glycosylation open search identified between 1,426 and 1,557 proteins (Fig. S2a), corresponding to 45.5%–52.9% of the total coding sequences (CDS). These proportions are consistent with the 48% reported in the earlier study (35). Throughout the growth phases, 1,245 proteins were consistently identified as core proteins, representing 80.0%–87.3% of the proteins identified. For *Np. piranensis*, between 1,209 and 1,387 proteins were identified (Fig. S2b), accounting for 57.2%–65.6% of CDS. The relatively higher ratio in *Np. piranensis* is probably due to its smaller genome size and fewer CDS. Throughout its growth phases, 1,091 proteins were consistently observed as core proteins, accounting for 78.7%–90.2% of identified proteins. These findings suggested that the protein profiles do not significantly differ across different growth phases. Many proteins encoded by the aforementioned *agl* genes were detected throughout the growth phases (Table 1), suggesting that protein *N*-glycosylation is consistently active across all growth phases in both species. However, it should be noted that NSAF values for oligosaccharyl transferase, a key enzyme in protein *N*-glycosylation, were relatively lower in *Np. piranensis* throughout growth phases (Table 1).

### Glycan and glycoprotein identification

**Fig 1:**
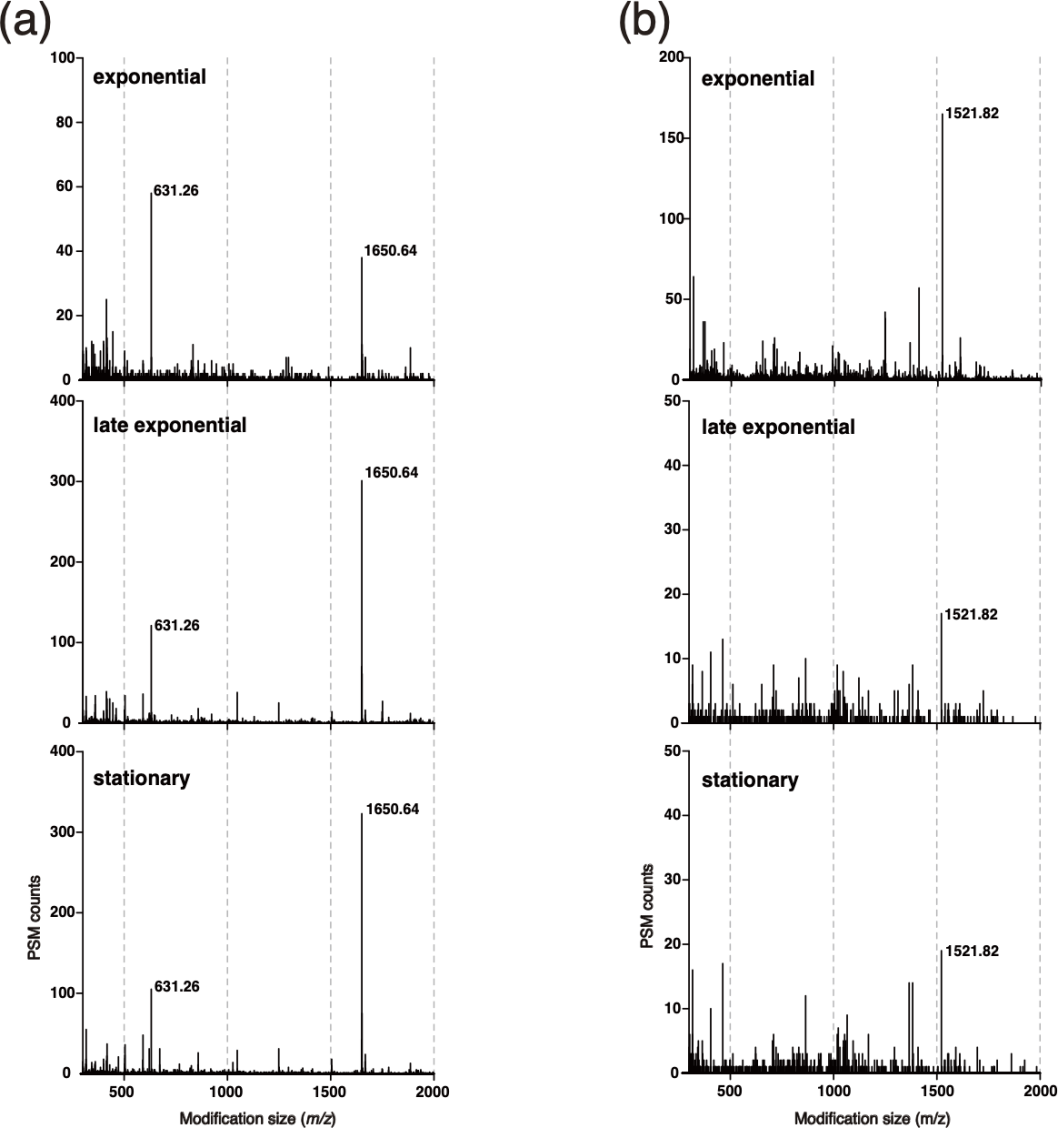
Modification mass distribution of glycopeptides in 0.1 Da increments, showing detected peptide-spectrum matches (PSMs) with modifications of *m*/*z* values ranging from 300 to 2,000 Da, derived from open searching data for *Ns. viennensis* (a) and *Np. piranensis* (b). Predominant *m*/*z* values are shown.

Peptide modification values obtained through Byonic open searching include both glycan modifications and random false values. To identify potential bona fide glycan modifications, we generated histograms of detected modification masses and evaluated their frequency distributions. This approach is effective in identifying the predominant *m*/*z* of glycans (54–56), especially since glycan heterogeneity within a single proteome is generally low in prokaryotes (67). For *Ns. viennensis*, histogram analysis revealed predominant modifications at 631.26 and 1,650.64 Da across all growth phases (Fig. 1a). These findings are in line with previous reports that indicate protein glycosylation remains stable across different growth phases in archaea (56). Peptide-spectrum match (PSM) counts, which indicate how often a peptide is identified in MS/MS spectra, increased for these modifications during the late exponential and stationary phases, suggesting they might represent a response to changes in surrounding environments, such as ammonium starvation. In the case of *Np. piranensis*, a distinct predominant modification at 1,521.82 Da was identified; however, its predominance was evident only during the exponential growth phase (Fig. 1B). Although the possibility that other less prevalent modification masses might correspond to minor glycans cannot be excluded, this analysis suggested significant differences in the glycosylation patterns between these archaea.

Given the demonstrated effectiveness of Byonic-focused searching for detecting glycopeptides and glycoproteins (54–56), we performed this analysis by targeting the predominant modification masses identified through open searching: 631.26 and 1,650.64 Da for *Ns. viennensis* and 1,521.82 Da for *Np. piranensis*. The glycoproteins confidently identified are detailed in Table 2. In *Ns. viennensis*, three different glycoproteins were identified, all undergoing *N*-glycosylation by the 1,650.64 Da glycan at asparagine residues within the conserved N-X-S/T sequon, a pattern common in archaea, bacteria, and eukaryotes (46), where X can be any amino acid except proline (Fig. S3). Although Byonic-focused searching suggested a putative modification at 631.26 Da in some proteins of *Ns. viennensis*, it was considered a false positive based on several criteria, including the absence of potential sugar oxonium ions in MS/MS spectra and the lack of the conserved N-X-S/T sequon. Details of the validation criteria are provided in Materials and Methods. These findings suggested that *Ns. viennensis* consistently exhibited only one predominant glycan modification on proteins throughout its growth phases, similar to glycosylation patterns observed in other archaea (54, 56).

**TABLE 2:** Glycoproteins identified in *Ns. viennensis* through focused search, including glycopeptide spectral matches (glycoPSMs), NSAF, and predicted localization^*a*^^,^^*b*^

Locus tag	Description	Localization	GlycoPSM			NSAF		
			Exponential	Late exponential	Stationary	Exponential	Late exponential	Stationary
NVIE_RS08145	Hypothetical protein (S-layer protein)	Cell wall	181	777	705	4.04	4.64	5.06
NVIE_RS00270	Multicopper oxidase domain-containing protein	Cytoplasmic membrane	24	42	81	2.74	2.82	4.23
NVIE_RS08635	Multicopper oxidase domain-containing protein	Unknown	11	12	3	0.13	0.14	0.09

^*a*^ Only proteins with a glycoPSM of 10 or more across any growth phases are included.

^*b*^ No glycoprotein was identified in *Np. piranensis*.

**Fig 2:**
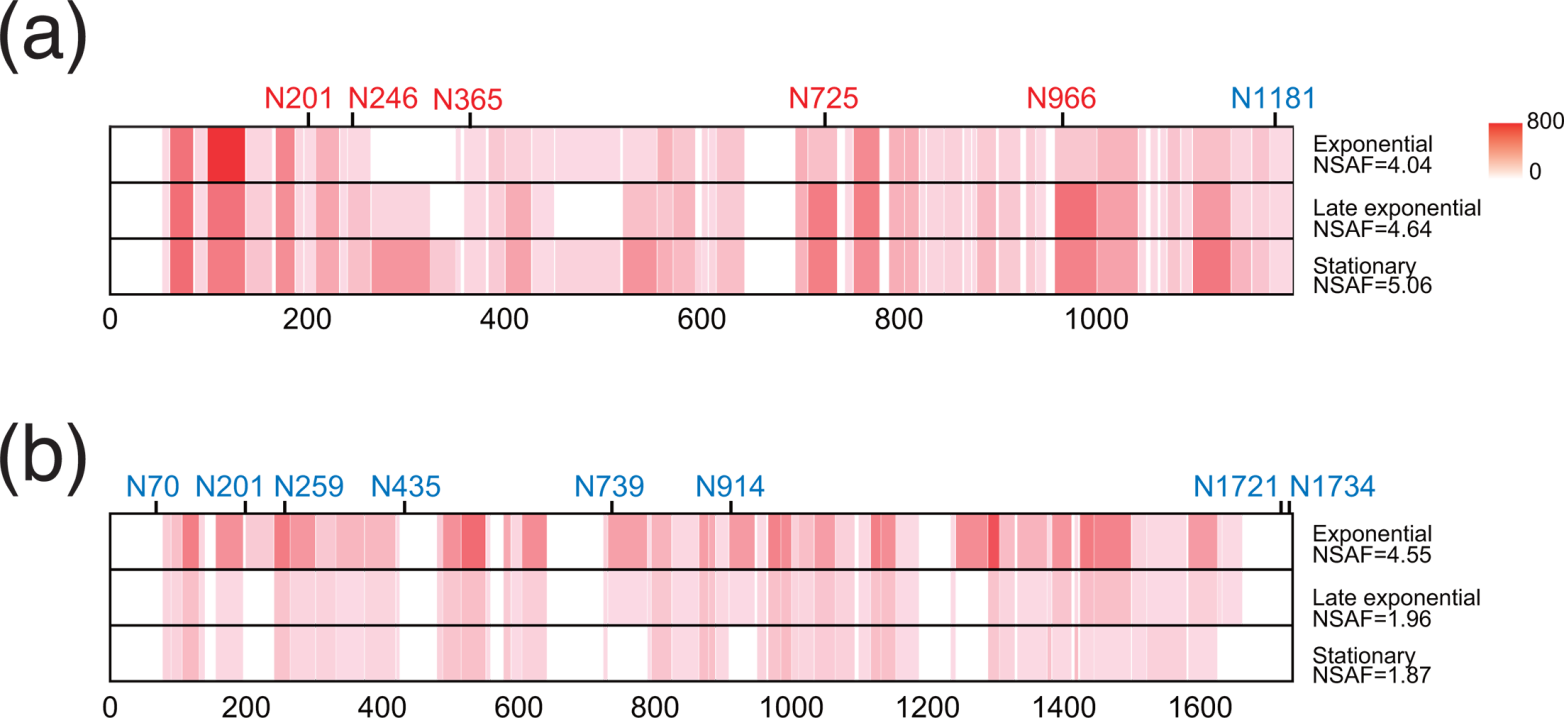
Heat maps visualizing the distribution of peptide-spectrum matches (PSMs) or glycopeptide spectral matches within the S-layer proteins of *Ns. viennensis* (a) and *Np. piranensis* (b). The numbers on the *X*-axis indicate amino acid positions starting from the N-terminus. The color intensity represents the frequency of peptide detection: an increased red intensity indicates higher PSM counts, on a scale from 0 to 800. Glycosylation sites where glycan modification was confirmed are highlighted in red, and those without confirmed glycosylation are shown in blue.

Although the protein encoded by NVIE_RS08145 (=NVIE_016740) in *Ns. viennensis*, which had the highest glycopeptide spectral match (glycoPSM) counts and NSAF values throughout growth phases (Table 2), was annotated as a hypothetical protein, it was identified as an S-layer protein in a previous study (35). Glycosylation of S-layer proteins is a common feature among diverse archaea (46). GlycoPP software predicted six potential *N*-glycosylation sites on this S-layer protein, including N1181; however, no glycosylation was detected at N1181 (Fig. 2a). Glycosylation was observed at the other five asparagine residues throughout the growth phases, with the highest total glycoPSM count occurring during the late exponential growth phase. The glycosylation frequency was one site per 239.8 residues, which is significantly lower than the frequencies observed for thermoacidophilic *Sulfolobales* spp., where one glycosite occurs every 30–40 residues (56, 68). The high frequency of *N*-glycosylation in *Sulfolobales* may be associated with the necessity of maintaining a stable and rigid cell wall under conditions of elevated temperatures and acidity in hot springs (46, 69). Conversely, the low frequency in *Ns. viennensis* may reflect the adaptation of *Nitrososphaera* to temperate soil habitats. Although glycoPSM counts and NSAFs were lower compared to the S-layer protein, other glycoproteins identified in *Ns. viennensis* were multicopper oxidase domain-containing proteins (MCOs) (Table 2). MCOs have been implicated in the oxidation of hydroxylamine to nitrite (1, 70–73). One of the glycosylated MCOs detected in *Ns. viennensis*, encoded by the gene NVIE_RS00270, was classified as nitrite reductase (NirK), which is typically involved in the reduction of NO_2_^−^ to NO. Although *N*-glycosylation in *N. maritimus* was suggested to be involved in protection from phages (45), the modification of both S-layer protein and MCOs by the same glycan highlights the importance of *N*-glycosylation for the energy metabolism of *Ns. viennensis*, potentially facilitating enzymatic processes crucial for nitrogen metabolism.

In contrast, no definitive protein *N*-glycosylation was detected in *Np. piranensis*, potentially due to the low abundance of oligosaccharyl transferase (Table 1). Similar to the 631.26 Da modification in *Ns. viennensis*, the 1,521.82 Da modification in *Np. piranensis* was also dismissed as a glycan modification. Although the S-layer protein encoded by the gene NPIRD3C_RS00110 of *Np. piranensis* harbored eight potential asparagine residues within the N-X-S/T sequon, no clear glycosylation evidence was observed, despite sufficient PSM counts (Fig. 2b). The Byonic-focused search identified a 1,521.82 Da modification on a specific peptide lacking an asparagine residue within the conserved sequon (PSMs = 301), and crucially, no potential sugar oxonium ions were detected in the MS/MS spectra. Although some asparagine residues, i.e., N70, N435, N1721, and N1734, were not assessed (Fig. 2b), this represents the first identification of a potentially non-*N*-glycosylated S-layer protein in archaea. This finding could indicate a distinct evolutionary or adaptive pathway in the glycosylation machinery of this archaeal species, potentially associated with genomic islands identified in marine AOA (74).

### *N*-Glycan structure of *Ns. viennensis*

**Fig 3:**
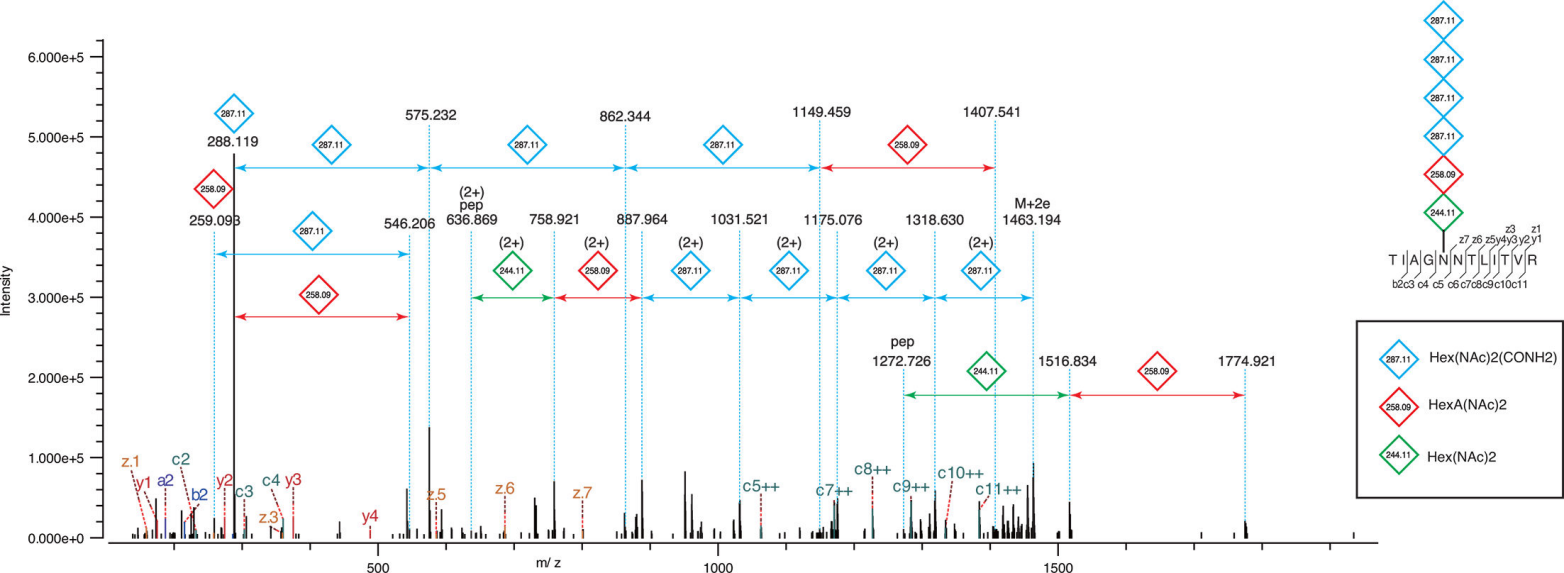
Electron-transfer/higher-energy collision dissociation MS/MS spectrum showing the *N*-glycan structure on the S-layer of *Ns. viennensis*. The spectrum displays the fragmentation pattern of the glycopeptide, including specific mass shifts corresponding to glycan or peptide constituents. The inset provides a diagrammatic representation of the glycopeptide, indicating probable glycan constituents attached at a specific asparagine residue.

**Fig 4:**
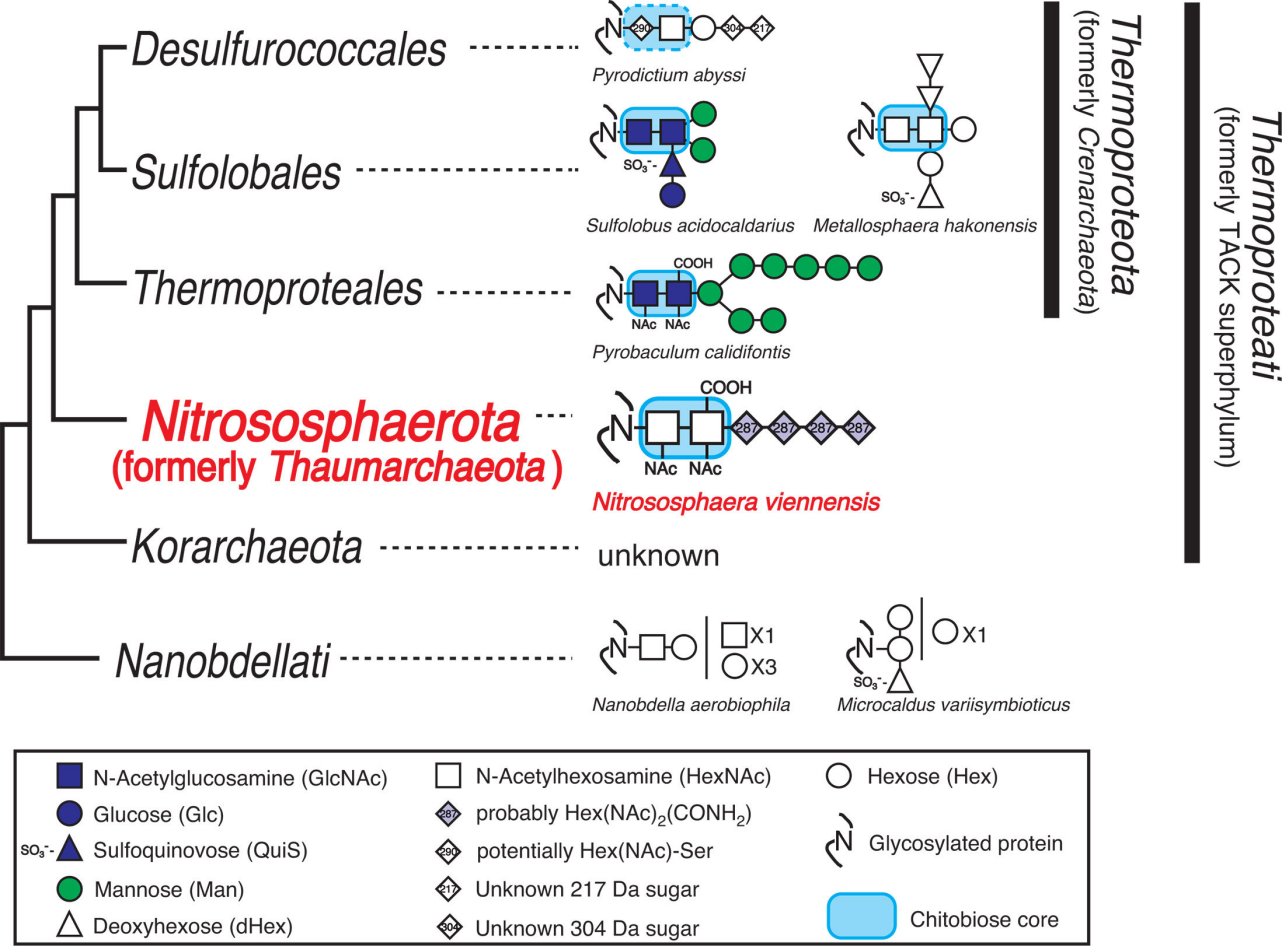
Structural diversity of *N*-linked glycans in archaea, showing examples from *Pyrodictium abyssi* (54), *Sulfolobus acidocaldarius* (52), *Metallosphaera hakonensis* (56), *Pyrobaculum calidifontis* (51), *Nanobdella aerobiophila* (56), and *Microcaldus variisymbioticus* (56).

The MS/MS analysis of glycopeptides from *Ns. viennensis* consistently revealed a hexasaccharide composed of atypical sugars. The examination of PSMs associated with the 1,650.64 Da modification mass uncovered a novel linear glycan structure composed of one di-*N*-acetylhexosamine [Hex(NAc)_2_], one di-*N*-acetylhexuronic acid [HexA(NAc)_2_], and four unknown sugar moieties each with a mass of 287.11 Da (Fig. 3). The linking sugar and its linked sugar, HexNAc with modifications, form a chitobiose core that resembles those found in members of *Thermoproteota* (formerly *Crenarchaeota*) (46), a characteristic also conserved in eukaryotes (Fig. 4). Notably, the Asn-Hex(NAc)_2_-HexA(NAc)_2_ configuration was previously reported in *Pyrobaculum calidifontis* (51), supporting the evolutionary link between the phyla *Nitrososphaerota* and *Thermoproteota* (2). Genome analysis has elucidated their evolutionary trajectory and associations with *Thermoproteota*, leading to their classification in the TACK superphylum (75), now recognized as the kingdom *Thermoproteati* (76). Previous studies have indicated that a chitobiose core of *N*-glycan is common among *Thermoproteota* archaea (51–54). Our findings suggest that this core structure originated from the common ancestor of the kingdom *Thermoproteati* (Fig. 4), providing new insights into the evolutionary origin of the core structure of eukaryotic *N*-glycans. Based on assumptions of a composition range of C_5–14_H_4–28_N_0–5_O_2–12_S_0–1_, a C/H ratio of 0–4, a C/NO ratio of 0.4–1.25, and an unsaturation range of 1–6 (67), our preliminary analysis suggested the unknown sugar moiety with a mass of 287.11 Da could potentially be C_11_H_17_O_6_N_3_ (theoretical mass = 287.1117).

**Fig 5:**
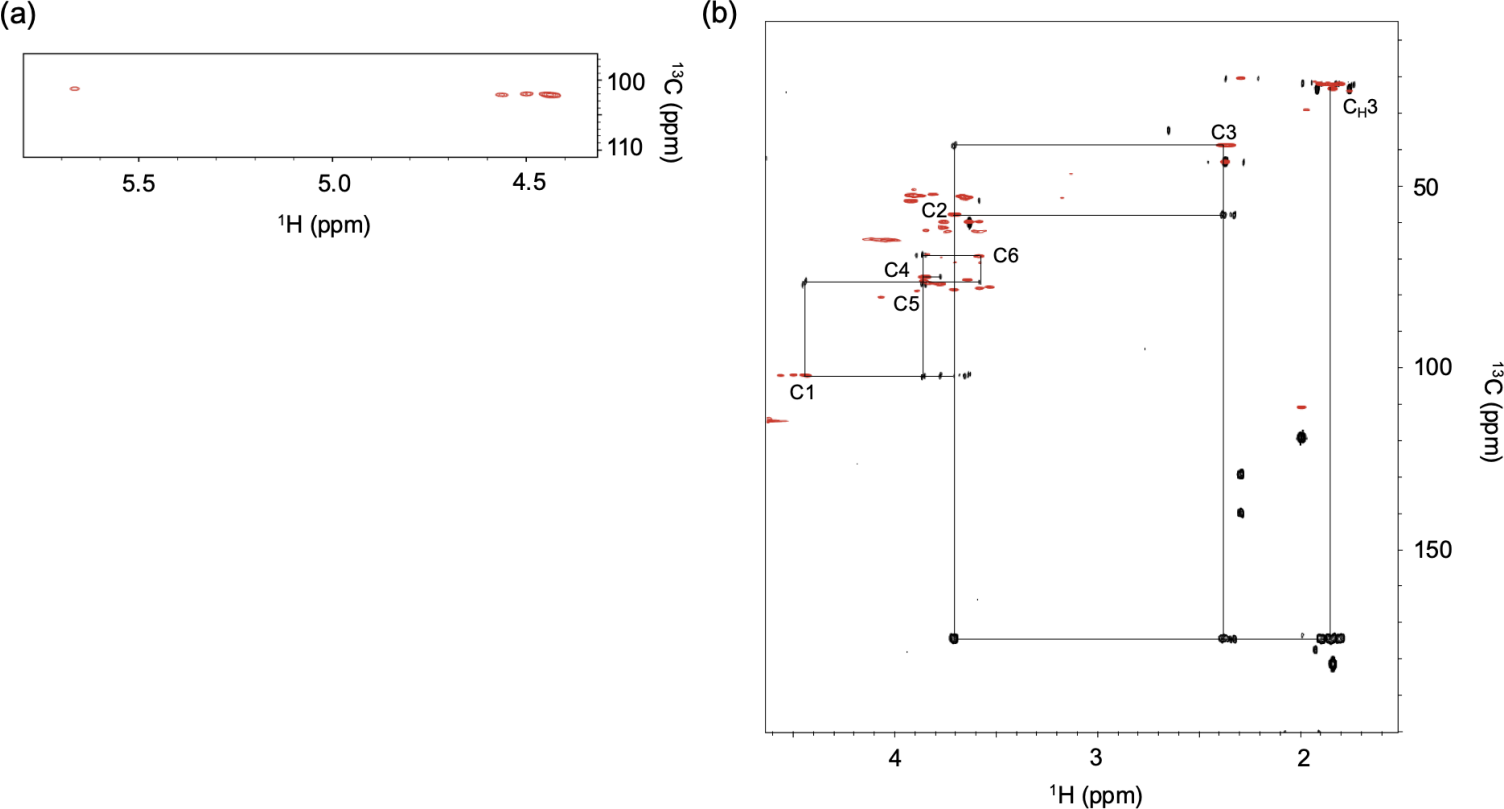
NMR spectra of the glycopeptide from *Ns. viennensis*. (a) The anomeric region of ^1^H-^13^C HSQC and (b) ^1^H-^13^C heteronuclear multiple bond correlation (black) and ^1^H-^13^C HSQC (red) spectra showing intraresidue scalar connectivities of the 287.11 Da units.

The anomeric region of the HSQC spectrum showed four peaks with an intensity ratio of 1:1:1:3 (Fig. 5a), indicating that the major peak corresponds to three overlapping signals from three equivalent units—specifically three of the four 287.11 Da residues, excluding the non-reducing terminal unit. These NMR data support the proposed composition formula and align with the MS results. Moreover, intraresidue scalar connectivities identified acetamide groups attached to C3 as well as C2 of the 287.11 Da unit (Fig. 5b), suggesting the presence of an additional functional group composed of C_1_H_2_N_1_O_1_, likely a carboxamide group. Consequently, the structure of the 287.11 Da unit is proposed as Hex(NAc)_2_(CONH_2_).

### Conclusion

Members of the phylum *Nitrososphaerota* are distributed globally across diverse oxic environments, with phylogenetically different members typically found in specific habitats (77). Phylogenetic analysis has elucidated the evolutionary trajectory of *Nitrososphaerota* members, suggesting that terrestrial AOA likely originated from non-AOA heterotrophic ancestors (38, 78). The ancestor of AOA is thought to have initially thrived in terrestrial geothermal environments, subsequently adapting to temperate soil habitats (39). Furthermore, AOA found in shallow marine environments, such as *Np. piranensis*, are considered to have evolved from terrestrial AOA, like *Ns. viennensis* (38, 39). This evolutionary sequence highlights the adaptive transitions of AOA from terrestrial to oceanic environments. Although proteins associated with *N*-glycosylation were detected in *Np. piranensis* as well, actual *N*-glycosylation was not observed in this study. This absence of detectable glycosylation could potentially be associated with the crystallographic characteristics of the S-layer (21) and the habitat, specifically being planktonic in the ocean rather than attached to solid surfaces in soil. The absence of detectable *N*-glycosylation in *Np. piranensis* despite the presence of glycosylation-associated proteins suggests a functional reduction of this modification system in this marine AOA. Given that phylogenetic analyses indicate marine *Nitrosopumilus* spp. have undergone extensive genome streamlining and adaptation to stable, low-temperature environments (79), it is plausible that *N*-glycosylation has been downregulated due to reduced selective pressure. Unlike terrestrial AOAs, which typically grow in aggregates and experience fluctuating environmental conditions that may favor robust cell surface modifications for biofilm formation (35, 40), marine AOAs thrive in relatively stable environments with a planktonic lifestyle and reduced cell-cell interactions, where the energetic cost of glycosylation may outweigh its benefits. Alternatively, the glycosylation machinery in *Np. piranensis* may be expressed under specific environmental conditions not replicated in our cultivation experiments. These findings highlight how cell surface modifications, including protein *N*-glycosylation, may have evolved differentially among AOA lineages in response to distinct ecological pressures.

Protein *N*-glycosylation plays a variety of roles in archaea (47, 80–82), particularly enhancing the structural stability of S-layers in extreme environments (46, 83). The detection of *N*-glycosylated S-layer proteins in the mesophilic and neutrophilic *Ns. viennensis* in this study pointed to different functions of S-layer glycosylation in archaea, potentially contributing to adaptation and survival in varied environmental conditions. Permanent protein adaptations are reflected at the amino acid sequence level and are designed to promote protein folding suitable for particular environments. In contrast, transient protein adaptations confer flexibility, allowing proteins to respond adaptively to changes in environments (84). Based on combined MS and NMR analysis, the structure of the *N*-linked hexasaccharide derived from *Ns. viennensis* is suggested to be novel, comprising Hex(NAc)_2_-HexA(NAc)_2_-Hex(NAc)_2_(CONH_2_)-Hex(NAc)_2_(CONH_2_)-Hex(NAc)_2_(CONH_2_)-Hex(NAc)_2_(CONH_2_). The two NAc groups in the Hex(NAc)_2_(CONH_2_) units are located at the C2 and C3 positions. By analogy, the NAc groups in the Hex(NAc)_2_ and HexA(NAc)_2_ residues are likely positioned similarly. Although a diverse range of *N*-glycan structures has previously been reported in archaea, this structure represents the most nitrogen-rich glycan identified so far in archaea (Fig. 4). Further analysis of other AOA and additional cultivation experiments are needed. However, the presence of two or three nitrogen-containing sugars could play a role in energy metabolism or nitrogen storage, particularly given the predominance of AOA in environments with low ammonium concentrations. This study provides new insights into a previously unrecognized diversity that may have implications for understanding their adaptive transitions to diverse environments.

## Data Availability

All liquid chromatography-tandem mass spectrometry raw files and complete protein identification lists have been deposited in the ProteomeXchange Consortium via jPOSTrepo (85), under the data set identifiers PXD056844 (JPST003423) for *Nitrososphaera viennensis* JCM19564 and PXD056846 (JPST003424) for *Nitrosopumilus piranensis* JCM32271.

## SUPPLEMENTAL MATERIAL

Supplemental Figures mbio.03859-24-s0001.docx Figures S1 to S3.

ASM does not own the copyrights to Supplemental Material that may be linked to, or accessed through, an article. The authors have granted ASM a non-exclusive, world-wide license to publish the Supplemental Material files. Please contact the corresponding author directly for reuse.
