# Supplementary material for: Exploring protein N-glycosylation in ammonia-oxidizing Nitrososphaerota archaea through glycoproteomic analysis
Source: mBio. 2025 May 19;16(6):e03859-24. doi: 10.1128/mbio.03859-24 (PMC12153357; doi:10.1128/mbio.03859-24)
Supplement: Supplemental Figures — Figures S1 to S3. [file mbio.03859-24-s0001.docx]

**Exploring protein *N*-glycosylation in ammonia-oxidizing *Nitrososphaerota* archaea through glycoproteomic analysis**

Satoshi Nakagawa^1,2,3^*, Hirokazu Yagi^3,4^, Tomoki Suyama^1^, Shigeru Shimamura^2^, Saeko Yanaka^3,5^, Maho Yagi-Utsumi^3,4,5^, Shingo Kato^6^, Moriya Ohkuma^6^, Koichi Kato^3,4,5^, Ken Takai^2,3^

^1^ Laboratory of Marine Environmental Microbiology, Division of Applied Biosciences, Graduate School of Agriculture, Kyoto University, Oiwake-cho, Kitashirakawa, Sakyo-ku, Kyoto 606-8502, Japan

^2^ Super-cutting-edge Grand and Advanced Research (SUGAR) Program, Institute for Extra-cutting-edge Science and Technology Avant-garde Research (X-star), Japan Agency for Marine-Earth Science and Technology (JAMSTEC), 2-15 Natsushima-cho, Yokosuka 273-0061, Japan

^3^ Exploratory Research Center on Life and Living Systems (ExCELLS), National Institute of Natural Sciences, 5-1 Higashiyama, Myodaiji, Okazaki, Aichi 444-8787, Japan

^4^ Graduate School of Pharmaceutical Sciences, Nagoya City University, 3-1 Tanabe-dori, Mizuhoku, Nagoya 467-8603, Japan.

^5^ Institute for Molecular Science (IMS), National Institutes of Natural Sciences, 5-1 Higashiyama, Myodaiji, Okazaki, Aichi 444-8787, Japan

^6^ Japan Collection of Microorganisms (JCM), RIKEN BioResource Research Center, Tsukuba, Ibaraki, Japan

Keywords: Glycan / *Nitrososphaerota* / Ammonia-oxidizing archaea / *Thaumarchaeota* / S-layer

*Corresponding author.

Phone: +81-75-753-6355; E-mail: nakagawa.satoshi.7u@kyoto-u.ac.jp


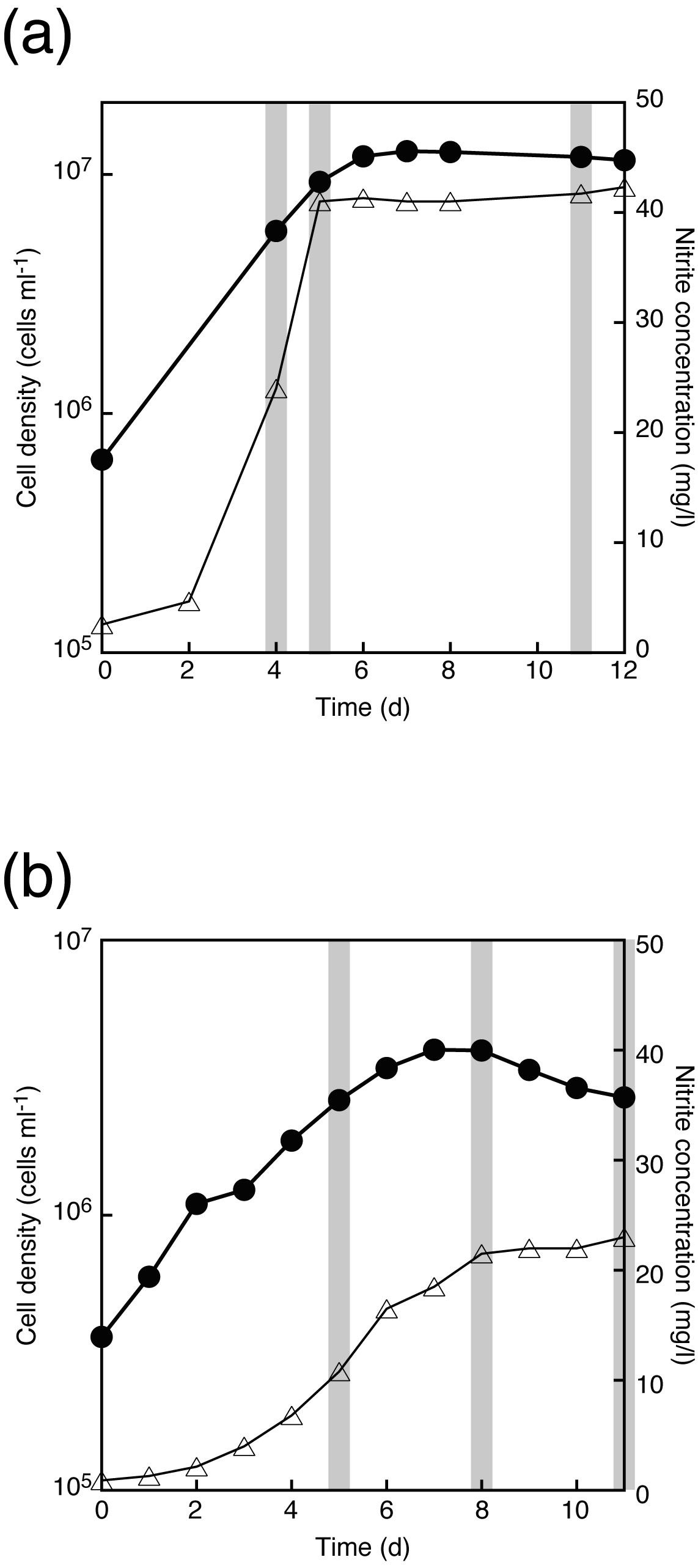


**Supplementary Figure S1.** Growth and nitrite production of *Ns. viennensis* (a) and *Np. piranensis* (b). Cells were harvested for glycoproteome analysis at points indicated by gray shading. Symbols: filled circle, cell density; open triangle, nitrite concentration.


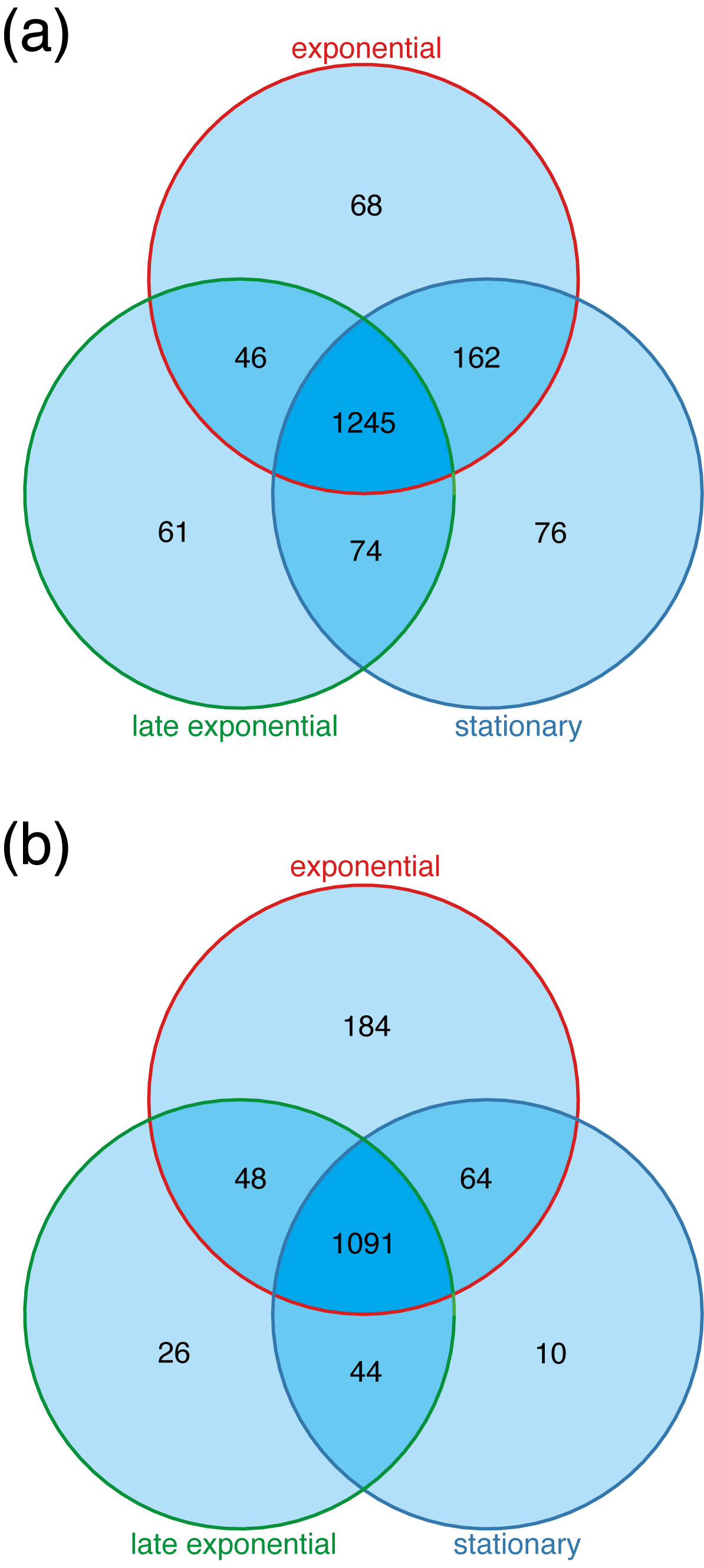


**Supplementary Figure S2.** Venn diagram illustrating the numbers of identified proteins shared among different growth phases of *Ns. viennensis* (a) and *Np. piranensis* (b).


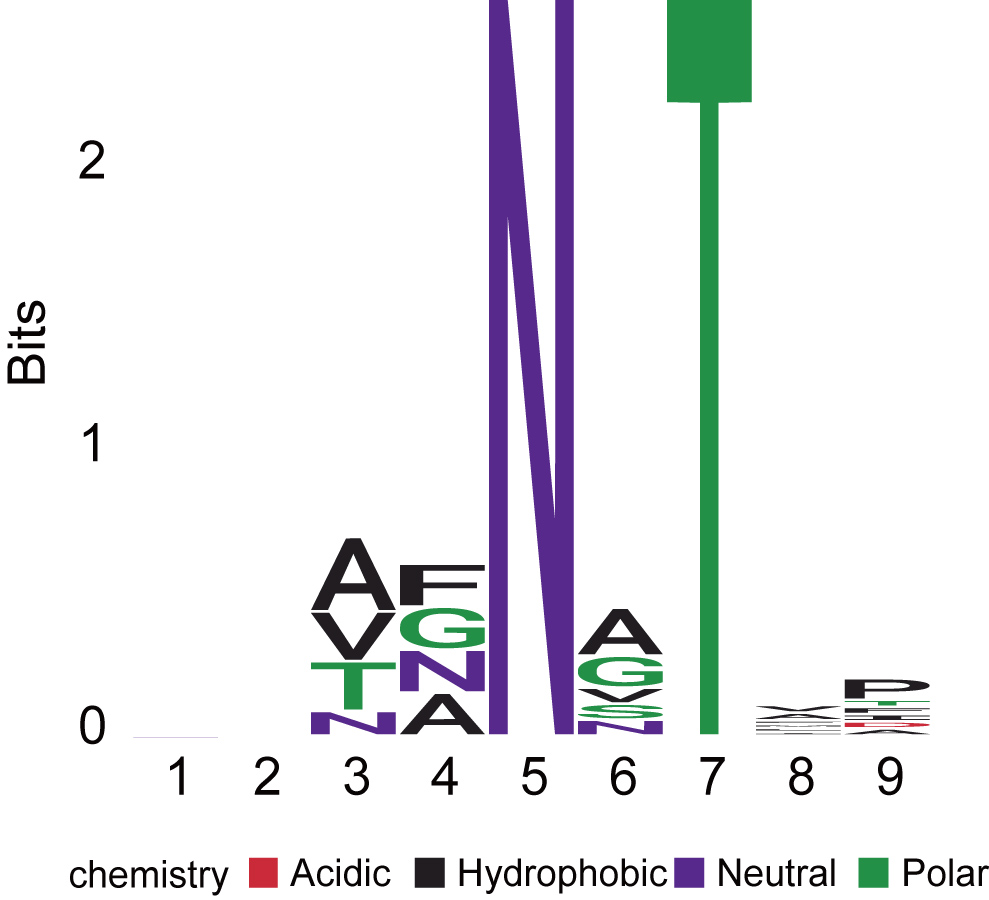


**Supplementary Figure S3.** Sequence logo representing consensus sequences for *N*-glycosylation sites on the three different glycoproteins in *Ns. viennensis*. Analysis was limited to glycopeptides shorter than 30 amino acids due to challenges in identifying *N*-glycosylation sites in longer peptides.
